# Preliminary Study on the Expression of Testin, p16 and Ki-67 in the Cervical Intraepithelial Neoplasia

**DOI:** 10.3390/biomedicines9081010

**Published:** 2021-08-13

**Authors:** Aneta Popiel, Aleksandra Piotrowska, Patrycja Sputa-Grzegrzolka, Beata Smolarz, Hanna Romanowicz, Piotr Dziegiel, Marzenna Podhorska-Okolow, Christopher Kobierzycki

**Affiliations:** 1 Division of Histology and Embryology, Department of Human Morphology and Embryology, Wroclaw Medical University, 50-368 Wroclaw, Poland; aleksandra.piotrowska@umed.wroc.pl (A.P.); piotr.dziegiel@umed.wroc.pl (P.D.); christopher.kobierzycki@umed.wroc.pl (C.K.); 2 Division of Anatomy, Department of Human Morphology and Embryology, Wroclaw Medical University, 50-368 Wroclaw, Poland; patrycja.sputa-grzegrzolka@umed.wroc.pl; 3 Department of Pathology, Polish Mother’s Memorial Hospital Research Institute, 93-338 Lodz, Poland; smolbea@wp.pl (B.S.); hanna-romanowicz@wp.pl (H.R.); 4 Department of Physiotherapy, University School of Physical Education, 51-612 Wroclaw, Poland; 5 Division of Ultrastructural Research, Wroclaw Medical University, 50-368 Wroclaw, Poland; marzenna.podhorska-okolow@umed.wroc.pl

**Keywords:** testin, p16 protein, cervical cancer, cervical neoplasia, immunohistochemistry

## Abstract

Cervical cancer is one of the most common malignant cancers in women worldwide. The 5-year survival rate is 65%; nevertheless, it depends on race, age, and clinical stage. In the oncogenesis of cervical cancer, persistent HPV infection plays a pivotal role. It disrupts the expression of key proteins as Ki-67, p16, involved in regulating the cell cycle. This study aimed to identify the potential role of testin in the diagnosis of cervical precancerous lesions (CIN). The study was performed on selected archival paraffin-embedded specimens of CIN1 (31), CIN2 (75), and CIN3 (123). Moderate positive correlation was observed between testin and Ki-67 as well as testin and p16 expression in all dysplastic lesions (r = 0.4209, r = 0.5681; *p* < 0.0001 for both). Statistical analysis showed stronger expression of the testin in dysplastic lesions vs. control group (*p* < 0.0001); moreover, expression was significantly higher in HSIL than LSIL group (*p* < 0.0024). In addition, a significantly stronger expression of testin was observed in CIN3 vs. CIN1 and CIN3 vs. CIN2. In our study, expression of Ki-67, p16, and testin increased gradually as the lesion progressed from LSIL to HSIL. The three markers complemented each other effectively, which may improve test sensitivity and specificity when used jointly.

## 1. Introduction

Cervical cancer is one of the most common malignant cancers in women worldwide. Each year, over 580,000 new cases of cervical cancer are diagnosed [1]. The American Cancer Society estimates that 4290 women will die due to cervical cancer in 2021 in the United States. In 2018, the number of deaths worldwide was 311,000, 90% of which occurred in low- and middle-income countries. The 5-year survival rate is 65%; nevertheless, it depends on race, age, and clinical stage. In developing countries in Africa, Asia, and South America, cervical cancer causes over 50% of early deaths in women of childbearing age. The WHO (World Health Organization) invests great effort into decreasing mortality and morbidity by means of promoting both primary and secondary preventive care for cervical cancer. In May 2018, WHO announced its project to eliminate cervical cancer as a public health issue between 2020 and 2030, reducing the age-correlated incidence rate to 4/100,000. WHO developed a triple intervention plan, which includes scaling up vaccination against HPV, twice-lifetime cervical screenings up to 70%, and treatment of preinvasive lesions and invasive cancer to 90% [2]. Informing patients that HPV is an important cause of cervical cancer has led to significant advances in the primary and secondary prevention of cervical cancer. Ten years ago, the WHO introduced vaccines against two of the most cancerogenic types, HPV16 and HPV18. They are highly effective at preventing HPV infections when administered before sexual activity. Nowadays there are available vaccines against seven carcinogenic HPV types and two non-cancer types that cause warts [3]. Cervical cancer is most often diagnosed in women aged 35–44, whereas it rarely occurs before 20 years of age. Over 15% of all cases occur in women above 65 years of age; however, it is rare in women who undergo regular screening. The main risk factor of cervical cancer is persistent HPV infection. It promotes impaired growth and differentiation of cells, leading to dysplasia (cervical intraepithelial neoplasia, CIN) [4]. We distinguished three grades of cervical dysplasia: CIN1 (LSIL; low-grade squamous intraepithelial lesion), CIN2, and CIN3 (classified together as HSIL; high-grade squamous intraepithelial lesion). The risk of developing invasive cervical cancer from HSIL is approximately 20% (10–40% according to literature) [5,6,7]. In the oncogenesis of cervical cancer, persistent HPV infection plays a pivotal role as it is necessary for CIN changes to occur. In assessing the risk of progression of lesions and the proper selection of treatment, techniques of viral DNA identification are used, which may be helpful in the triage of patients with cytological diagnosis of atypical squamous cells of undetermined significance (ASCUS). HPV DNA tests are highly sensitive, but the specificity of HPV tests is low due to most HPV infections being naturally cleared [8]; this creates the need to search for more specific diagnostic methods. More specific markers of cervical cancer have been identified from HPV-induced oncogenesis studies. The oncogenic HPV viruses disrupt the expression of key proteins as Ki-67, p16 involved in regulating the cell cycle. Our study aimed to identify the potential role of testin protein in the diagnosis of cervical precancerous lesions. Research points to its possible role in cervical cancer, whereas in CIN it has not been analyzed yet [9,10]. In this article, we focus on the correlation between testin and known markers used in the diagnosis of cervical intraepithelial changes.

## 2. Materials and Methods

### 2.1. Material

The study was performed on selected archival paraffin-embedded specimens of CIN1 (31), CIN2 (75), and CIN3 (123). Patients aged from 25 to 86 years old were female (Table 1). The control group consisted of 125 cases of normal cervical tissue possessed from patients who underwent total hysterectomy due to uterine leiomyomas. Patients were operated on between 2014 and 2017 in the Polish Mother’s Memorial Hospital in Lodz.

### 2.2. TMA Construction

Hematoxylin and eosin-stained (HE) 6-μm thick paraffin sections were prepared to verify the histopathological diagnosis and assess the suitability of the sample for further analysis. In short, slides were scanned utilizing histologic scanner Pannoramic MIDI (3DHistech Ltd., Sysmex Suisse AG, Horgen, Switzerland). Afterward, scans were examined by two independent pathologists who chose and electronically labeled areas of the CIN in the changed epithelium of the cervix. For TMA construction, from the corresponding paraffin donor blocks, triplicate tissue core punches (2 mm) for every case were obtained (TMA Grand Master; 3DHistech, Budapest, Hungary). The normal epithelial tissue of the cervix was marked as a control group.

### 2.3. Immunohistochemistry (IHC)

Immunohistochemical reactions were performed on 4 μm paraffin sections obtained from TMA blocks in an automated staining platform, Autostainer Link48 (Dako, Glostrup, Denmark). Deparaffinization, rehydration, and antigen retrieval was performed using EnVision FLEX Target Retrieval Solution (97 °C, 20 min; pH 6 for Ki-67 and pH 9 for p16 and testin) in PT-Link. The activity of endogenous peroxidase was blocked by 5 min exposure to a peroxidase-blocking reagent (Dako). Monoclonal mouse anti-p16 antibody (1:100+linker, 550834, BP Pharmingen, San Diego, CA, USA), anti-Ki-67 (ready to use, IR626, Dako), and polyclonal rabbit anti-testin (1:400, NBP1-87987, Novus Biologicals, Centennial, CO, USA) were used as the primary antibody (20 min incubation) followed by incubation with a secondary antibody conjugated with horseradish peroxidase (EnVision™ FLEX/HRP—20 min incubation). 3,3′-diaminobenzidine (DAB) was utilized as the peroxidase substrate, and the sections were incubated for 10 min. Finally, all sections were counterstained with EnVision FLEX Hematoxylin (Dako) for 5 min. After dehydration in graded ethanol concentrations (70%, 96%, absolute) and xylene, all slides were closed with coverslips in SUB-X Mounting Medium in a coverslipper.

The slides were scanned using a histologic scanner, Pannoramic MIDI (3DHistech). Reactions were evaluated (Ki-67) with the use of Quant Center software (3DHistech) under researcher supervision. For every case, three TMA cores were quantified by the algorithm SCORE (range = 0–8), and the final result was an average count. Expression of testin was assessed using a Pannoramic Viewer Digital image analysis as well as a routinely used immunoreactive scale (IRS) by Remmele and Stegner, presented in Table 2. The expression of p16 antigen was evaluated by two parameters, a percentage of p16-positive cells, and reaction intensity. The percentage of positive cells was evaluated in the highest expression area (‘‘hot spot’’) and graded as follows (grade 0) when no cells stained, positive cells >0–5% (grade 1), positive cells >5–25% (grade 2), positive cells >25% (grade 3). The intensity of the reaction was scored as negative (0), weak (1), moderate (2), and strong (3). The reaction was considered as positive when nuclear, or nuclear and cytoplasmic, strong, and diffuse p16 staining beginning from the basal cell layer of the epithelium was observed. Whereas non-specific patterns, focal, wispy, small clusters of cells and a complete lack of staining qualified as negative p16 expression. Testin cytoplasmatic expression was scored as follows:

### 2.4. Statistical Analysis

The results were statistically analyzed using GraphPad Prism 5.0 software using Spearman correlation, Kruskal–Wallis, Dunn’s multiple comparison, and Mann–Whitney tests. In all analyzed cases, the associations were considered statistically significant for *p* < 0.05.

## 3. Results

**Figure 1:**
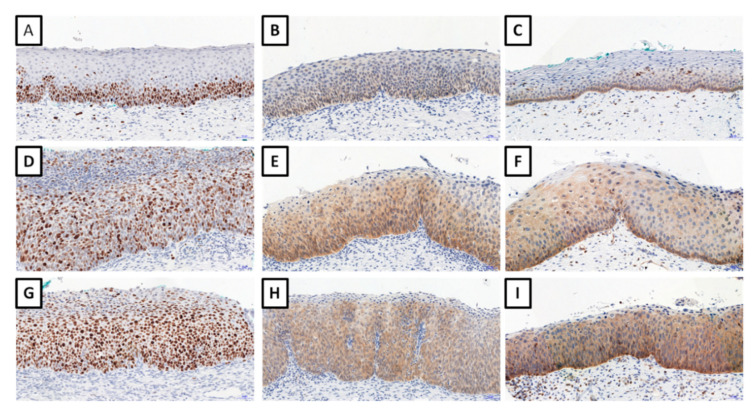
Immunohistochemical expression of Ki-67 ((**A**), CIN1; (**D**), CIN2; (**G**), CIN3); p16 protein ((**B**), CIN1; (**E**), CIN2; (**H**), CIN3), and testin ((**C**), CIN1; (**F**), CIN2; (**I**), CIN3). Magnification ×200.

Expression of Ki-67 was observed in 100%, p16 in 84.6%, and testin in 98.25% of cervical dysplasia cases (Figure 1).

A moderate positive correlation was observed between testin and Ki-67 as well testin and p16 expression in all dysplastic lesions (r = 0.4209, r = 0.5681; *p* < 0.0001 for both; Spearman correlation test). The relationships subdivided according to CINs are presented in Table 3.

**Figure 2:**
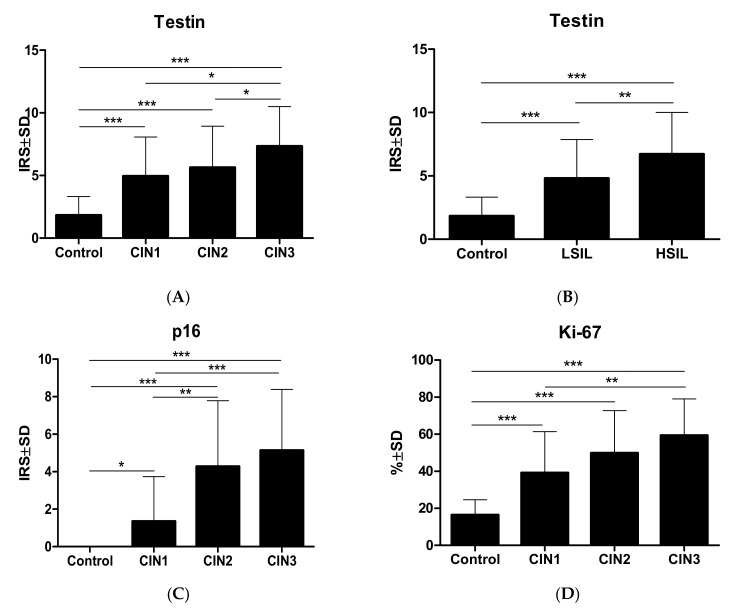
Immunohistochemical reaction in cervical intraepithelial neoplasia: (**A**) testin expression in CIN lesions; (**B**) testin expression in LSIL and HSIL lesions; (**C**) p16 expression in CIN lesions; (**D**) Ki-67 expression in CIN lesions. * *p* < 0.05, ** *p* < 0.01 and *** *p* < 0.001.

**Figure 3:**
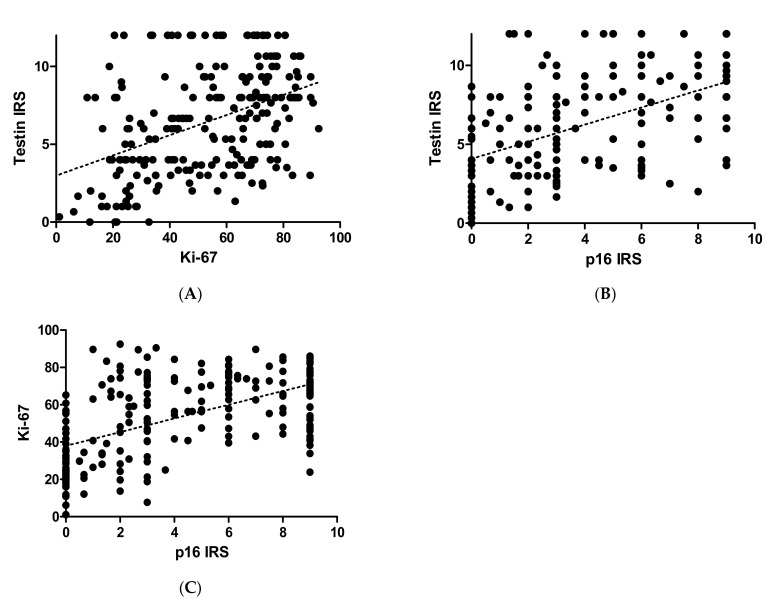
Immunohistochemical reaction in cervical intraepithelial neoplasia: (**A**) testin and Ki-67 correlation in CIN lesions; (**B**) testin and p16 correlation in CIN lesions; (**C**) Ki-67 and p16 correlation in CIN lesions.

**Figure 4:**
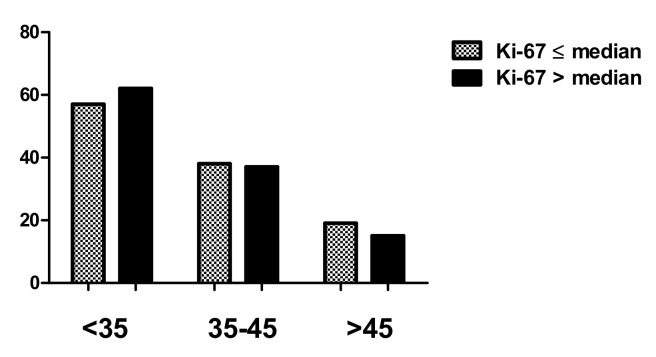
Median of the Ki-67 expression in age groups of CIN lesions.

**Figure 5:**
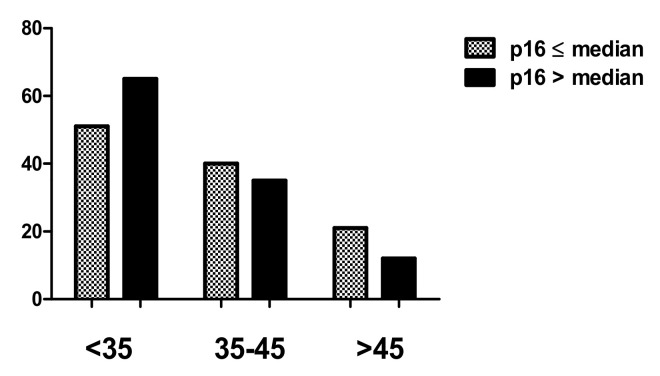
Median of the p16 expression in age groups of CIN lesions.

Statistical analysis showed stronger expression for testin in dysplastic lesions vs. control group (*p* < 0.0001; Mann–Whitney test; Figure 2A). Moreover, the expression was significantly higher in HSIL than the LSIL group (*p* < 0.0024; Mann–Whitney test; Figure 2B). Expression of p16 and Ki-67 was stronger in all dysplastic lesions vs. control group (*p* < 0.0001; Mann–Whitney test; Figure 2 C,D), and the expression of these two markers was higher in HSIL than in LSIL (*p* < 0.0001; Mann–Whitney test). The expression of p16 does not show statistically significant differences between normal cervical tissue and CIN1 (*p* > 0.05; Dunn’s multiple comparison test). The expression of Ki-67 in normal cervical tissue was significantly lower than in CIN1 (*p* < 0.05; Dunn’s multiple comparison test). In addition, significantly stronger expression of testin was observed in CIN3 vs. CIN1 and CIN3 vs. CIN2 cases (*p* < 0.05 respectively; Dunn’s multiple comparison test). There were no statistically significant differences in testin expression between CIN1 and CIN2 as well as in p16 expression between CIN2 and CIN3 (*p* > 0.05; Dunn’s Multiple Comparison test). Additionally, moderate positive correlation was observed between the expression of testin and Ki67, testin and p16 also between Ki-67 and p16 in all dysplastic lesions (*p* < 0.0001, r = 0.3917; *p* < 0.0001, r = 0.5681; *p* < 0.0001, r = 0.5655 Spearmann correlation test; Figure 3A–C). No differences in the relationship between age groups and type of CIN were observed. The highest percentage of HSIL lesions occurred in the <35 age group (Table 4). In addition, there was no statistically significant relationship between age groups and Ki-67 (Figure 4) or p16 median (Figure 5), but the relationship between age group and testin was close to being statistically significant (Chi-square test; *p* = 0.0667; Table 5).

## 4. Discussion

The term dysplasia refers to the replacement of normal squamous stratified epithelium by abnormal cells with pathological morphology spreading on successive layers of the epithelium. In the early 1980s, this term was changed into cervical intraepithelial neoplasia by the International Society of Gynecological Pathologists [11]. The terminology change was dictated by a detailed understanding of cervical cancerogenesis. All changes in the epithelium leading to the formation of CIN1, CIN2, CIN3, and cervical cancer are a series of related events, not isolated actions as previously believed. For clinicians, the most important aspect of diagnostics for CIN is to differentiate LSIL from HSIL. For this reason, many currently ongoing studies search for new markers that may streamline the diagnostic process. In this study, we focused on the correlation between testin and Ki-67 as a marker of proliferation and p16, indicating the impact of the HPV infection on the cervical epithelium. The latest report indicates that the TES gene is a tumor suppressor gene that can contribute to cancerogenesis, but the mechanism of the loss of TES gene expression is still unknown. HPV infection leads to the overexpression of E6 and E7 oncoproteins which disrupt the normal function of the tumor suppressor gene [12]. There is a lack of studies that analyze the expression of testin in LSIL and HSIL. The expression of this producer in CIN may prove an important factor in the development of cervical lesions.

P16, also known as p16INK4a, is encoded by the cyclin-dependent kinase inhibitor 2A (CDKN2A) gene located on chromosome 9p21.3. It is a cell cycle protein that regulates cell proliferation in the G1-S phase due to the reciprocal relationship with another tumor suppressor protein-Rb. In persistent HPV infection, oncogenic proteins E6 and E7 bind to host regulatory proteins. In HPV, E6 oncoproteins lead to the dysfunction of the p53 suppressor gene through direct protein–protein interaction, inducing p53 protein degradation [13]. On the other hand, E7 oncoproteins form a complex with retinoblastoma (Rb) protein that blocks the phosphorylation of Rb protein, thereby increasing free E2F; this results in both abnormal cell cycle progression and overexpression of p16 protein. Overexpression of p16 is commonly found in cells infected by HPV. Currently, IHC expression of p16 together with the expression of Ki-67 is routinely used to improve diagnosis of cervical lesions [14,15,16]. In the present study, the correlation between the grade of cervical lesions and the expression of p16 was strong (*p* < 0.0001). A similar correlation was observed according to the expression of Ki-67. It directly confirms the observation of other authors’ research and indicates the validity of using commercial kits (CINtec PLUS Kit) to diagnose LSIL and HSIL [17,18,19,20]. Our study confirms the results of other research and indicates that p16 expression positively correlates with the degree of cervical lesions [21]. This study also shows no statistically significant difference between the expression of p16 in normal cervical tissue and CIN1, suggesting that p16 does not fully reflect the degree of cervical lesions. The absence of p16 expression can be used to eliminate associated high-grade squamous intraepithelial lesions in biopsy material. There is one limitation of p16 analysis as a CIN marker. P16 expression can sometimes be focal or diffuse in benign endocervical intercalated columnar cells, tubal metaplasia of the endometrium, and cervical endometriosis [22]. Despite that, the expression of p16 in these cells does not have the premalignant potential [23].

Ki-67 is a cellular marker of proliferation, detected in the non-G0 phase of the cell cycle [24]. In normal squamous cervical epithelium, Ki-67 is present only in basal and parabasal layers. In dysplasia and carcinoma, their expression extends above the basal one-third of squamous epithelium [25]. Many studies have shown that the elevated expression of Ki-67 is closely related to cell mitosis and cell proliferation [26,27,28,29]. Some scientists highlight this marker’s role in distinguishing different degrees of cervical lesion [21,28,29]. The results of this study showed that the expression of Ki-67 was higher in the CIN1, CIN2, and CIN3 groups than in the control group (*p* < 0.05). The expression level of Ki-67 was significantly higher in HSIL than LSIL (*p* < 0.05), indicating that the expression level increased with the development of the HSIL lesions. Moreover, there is a significant difference in Ki-67 expression between normal cervical tissue and CIN1. Some studies show that Ki-67 is expressed in proliferative non-cancerous tissue, and in this study, patients with LSIL also have a positive expression of Ki-67 [30]. High expression of Ki-67 is associated with the severity of cervical lesions but not with HPV infection [31]. There is evidence that Ki-67 has a prognostic value superior to the standard histopathological grading to prognosticate CIN progression. Several studies have shown that diffuse expression of Ki-67 is present in almost all cases of HSIL or cervical cancer [32,33,34].

Testin is a protein with molecular mass 47kDa. The human TES gene is localized on the fragile site FRA7G at 7q31.2 [35]. Testin protein comprises three LIM (Linl-1, Isl-1, Mec-3) domains, and each consists of two zinc fingers linked by two amino acid spacers [36]. It was observed that the N- and C-terminal parts could interact with each other and create open and close conformations in cells. When expressed separately in cells, these two halves show partially different subcellular localizations with a dominant role of LIM domains targeting focal adhesion. Testin is localized along the actin stress fibers at the cell–cell junction and focal adhesion. Testin can interact with cytoskeletal proteins such as zyxin, talin, and VASP [9,37]. Together, they play a significant role in cell motility and adhesion. In chicken and human fibroblast, testin notably activates cell spreading, but there is a loss of testin increased cell motility. These suggest that testin appears crucial in regulating cellular migration, invasion, and process of epithelial-mesenchymal transition. Additionally, testin is involved in the cell cycle. The expression of testin protein positively correlates with a percentage of cells in the G1 phase; however, overexpression can induce apoptosis and decreased colony-forming ability. The expression of testin has been described in many types of human malignancies, but there is fewer data about the expression of testin in cervical intraepithelial neoplasia. The mechanism and pathways of testin’s influence on cancerogenesis are still unknown. Some of the authors point out hypermethylation of the gene as the main factor. [35,36,38,39,40,41,42,43]. In this study, the expression of testin was significantly stronger in all dysplastic lesions compared to the control group. Testin expressed stronger in HSIL than LSIL; this indicates testin as a good diagnostic marker for distinguishing cervical lesions (CIN1 vs. CIN2 and CIN3). Zhong et al. support that testin has open and close conformations in cells by detecting the anti-TES serum in the nucleolus and anti-TESC as well anti-TESN sera in the cytoplasm. Cellular location is important for protein function because many phosphates, kinases, and transcription factors regulate their activity by controlling subcellular distribution [44]. Testin shuttling among cellular compartments may have divergent functions with different interacting partner proteins. The multiple conformational states and different locations in cellular compartments impact various expressions of testin protein; this may be the factor that suggests low testin expression in HSIL lesions. Future studies should be performed to find functions correlating with different cellular locations [45]. We plan on expanding our research with further studies to include cancer tissue. We plan on researching correlations between testin and lymphovascular space invasion, nodes metastasis, angiogenesis, and epithelial-mesenchymal transition markers in the upcoming months.

## 5. Conclusions

In our study, the expressions of Ki-67, p16, and testin gradually increased as the lesion progressed from LSIL to HSIL. The three markers complemented each other effectively, which may improve test sensitivity and specificity when used jointly.

## Figures and Tables

**Table 1:** Clinicopathological features of study group patients.

Feature	N	%
Age		
<35	119	51.97
35–45	75	32.75
>45	35	15.28
Cytology result		
CIN 1	31	13.5
CIN 2	75	32.8
CIN 3	123	53.7
Histology result		
LSIL	31	13.5
HSIL	198	86.5

**Table 2:** Immunoreactive scale scoring system.

Score	Positively Stained Cells (PP)	Intensity of Staining (SI)	IRS Points (PP × SI)	IRS Classification
**0**	no staining	no color reaction	0–1	Negative
**1**	<10%	mild reaction	2–3	Positive, mild expression
**2**	10–50%	moderate reaction	4–8	Positive, moderate expression
**3**	51–80%	strong reaction	9–12	Positive, strong expression
**4**	>80%			

**Table 3:** Spearman correlation test results.

	CIN1		CIN2		CIN3	
	Ki-67	p16	Ki-67	p16	Ki-67	p16
**testin**	r = 0.3678 *p* < 0.0418	NS	r = 0.3441 *p* < 0.0025	r = 0.6348 *p* < 0.0001	r = 0.3406 *p* < 0.0001	r = 0.4364 *p* < 0.0001
**Ki-67**		r = 0.5342 *p* < 0.0028		r = 0.6032 *p* < 0.0001		r = 0.3802 *p* < 0.0001

**Table 4:** LSIL and HSIL in specific age groups.

	LSIL	HSIL
**<35**	13	106
**35–45**	13	62
**>45**	5	30

**Table 5:** Median of the testin expression in age groups of CIN lesions.

	Testin < Median	Testin > Median
**<35**	58	61
**35–45**	49	26
**>45**	18	17

## Data Availability

The data presented in this study are available upon request from the corresponding author. The data are not publicly available due to privacy issue.
